# An In Vitro Investigation of Cytotoxic Effects of InP/Zns Quantum Dots with Different Surface Chemistries

**DOI:** 10.3390/nano9020135

**Published:** 2019-01-22

**Authors:** Deanna Ayupova, Garima Dobhal, Geoffry Laufersky, Thomas Nann, Renee V. Goreham

**Affiliations:** 1 MacDiarmid Institute for Advanced Materials and Nanotechnology, Wellington 6140, New Zealand; deanna.ayupova@vuw.ac.nz (D.A.); garima.dobhal@vuw.ac.nz (G.D.); geoffry.laufersky@vuw.ac.nz (G.L.); thomas.nann@newcastle.edu.au (T.N.); 2 Centre for Biodiscovery, Victoria University of Wellington, Wellington 6012, New Zealand; 3 School of Chemical and Physical Sciences, Victoria University of Wellington, Wellington 6012, New Zealand

**Keywords:** quantum yields, aptamer, photoluminescence, cytotoxicity, quantum dots, bioimaging

## Abstract

Indium phosphide quantum dots (QDs) passivated with zinc sulphide in a core/shell architecture (InP/ZnS) with different surface chemistries were introduced to RAW 264.7 murine “macrophage-like” cells to understand their potential toxicities. The InP/ZnS quantum dots were conjugated with an oligonucleotide, a carboxylic acid, or an amino-polyethylene glycol ligand, and cell viability and cell proliferation were investigated via a metabolic assay. Membrane integrity was measured through the production of lactate dehydrogenase. Fluorescence microscopy showed cellular uptake. All quantum dots exhibited cytotoxic behaviour less than that observed from cadmium- or lead-based quantum dots; however, this behaviour was sensitive to the ligands used. In particular, the amino-polyethylene glycol conjugated quantum dots proved to possess the highest cytotoxicity examined here. This provides quantitative evidence that aqueous InP/ZnS quantum dots can offer a safer alternative for bioimaging or in therapeutic applications.

## 1. Introduction

Quantum dots (QDs) are semiconductor nanocrystals that show high optical brightness, narrow emission wavelength, and increased photostability compared with organic fluorophores. This makes them ideal markers for bio-imaging applications such as tumour diagnostics and therapeutics [1]. Unlike work using molecular probes, research using QDs exploits resistance to photobleaching, which allows for their use in high-intensity and long-term bioimaging [2,3]. Another advantage of QDs is for real-time cell tracking, allowing for the visualisation of extracellular movement. As QDs can be functionalised with antibodies or aptamers (targeting nucleotide ligands), they can be used to specifically identify cellular proteins or other moieties. Despite the above advantages, there is concern regarding the cytotoxic behaviour of the nanomaterials, in particular, the inherently toxic cadmium (Cd) and lead (Pb)-based QDs. Several studies have shown that Cd-based QDs can cause damage to cells and significant DNA damage due to acute toxic effects [4,5,6,7]. QDs are considered high-risk respiratory hazards due to their small size, which allows them to reach lung alveoli [6,8]. To combat these effects, less toxic semiconductor shells, such as zinc sulphide (ZnS), can be grown on the core QD material, reducing overall cytotoxicity of the particles [9]. The degradation and cytotoxic effects of core/shell QDs can be further reduced by strictly controlling factors such as the size and the surface ligand chemistry of the particles. Despite these improvements, there are still widespread concerns over the chronic effects of even statistically minor toxic events over the course of the lifetime of an individual, which significantly reduces the large scale viability of these products [10,11].

Indium phosphide (InP) nanomaterials display many of the same advantages in size, resistance to photobleaching, and tunable fluorescence compared to their Cd-based counterparts but are less toxic due to their constituent materials. As such, core/shell structured InP/ZnS QDs have become popular alternatives to cadmium and lead nanomaterials. However, despite their theoretical advantages, their overall cytotoxic behaviour is still poorly understood [12,13,14]. To date, most studies present only colorimetric assays for assessing cell metabolic activity (such as the MTT assay), which do not provide the full scale of cellular responses upon QD exposure. Overall, InP/ZnS have demonstrated quality evidence for biocompatibility but further research is necessary to provide a complete picture of their cytotoxicity.

In this study, we demonstrated the modification and characterisation of InP/ZnS QDs with different surface chemistries. Three common ligands used in the biofunctionalisation of nanomaterials were attached to the QDs: a 30-mer oligonucleotide (Oligo), mercaptosuccinic acid (MSA), and an amine terminated polyethylene glycol (PEG-NH_2_; average Mn = 5000 g/mol). MSA-functionalised QDs showed less cytotoxicity in all concentrations compared to the other ligands, while those conjugated with PEG-NH_2_ demonstrated both a greater cellular uptake and higher cytotoxicity. It was also demonstrated that oligonucleotides could be conjugated to the InP/ZnS QDs which can then be used to target a specific moiety, such as a surface protein on a cell. Recent advances of aptamer-functionalised QDs were summarised by Wen et al. with prevalence of optical and fluorescent advantages [15]. Our cytotoxicity studies show that QD-Oligo conjugates revealed a lower level of cytotoxicity and membrane disruption, as well as membrane cellular uptake investigated via Alamar Blue assay, lactate dehydrogenase (LDH) release, reactive oxygen species (ROS) detection, TNFα secretion, and confocal fluorescent microscopy. This work demonstrates the low cytotoxicity of InP/ZnS QDs compared to more toxic QDs. Additionally, the ability to perform ligand exchange for custom designed QDs provides more applications in bioimaging or biosensor platforms.

## 2. Materials and Methods

### 2.1. Methods

All statistical analyses are presented as the mean ± standard error of mean (SEM). Statistical significance was determined by using one-way analysis of variance (ANOVA) (Prism 7 software, version 7.0a, GraphPad, San Diego, CA, USA). The results were considered significant if *p* ≤ 0.05.

### 2.2. Materials

All chemicals and reagents were purchased from Sigma-Aldrich (St. Louis, MO, USA), unless stated otherwise. InP/ZnS QD synthesis and ligand exchange procedures and characterisation have been detailed in the Supplementary Information.

### 2.3. Cell Culture

RAW 264.7 murine “macrophage-like” cells with mouse monocyte macrophage cell line origin were obtained from the American Type Culture Collection (ATCC). Cells were cultured in complete growth medium with the following components with a base of formulated Dulbecco’s Modified Eagle’s Medium (DMEM) cell culture medium containing 4.5 g/L D-glucose, 110 mg/L Sodium Pyruvate, no L-Glutamine (Gibco™, cat.10313201), heat-inactivated foetal bovine serum (FBS) to a final concentration of 10% (Sigma, St. Louis, MO, USA), and 1% Penicillin/Streptomycin (P/S) (Gibco™, cat.15140163) in a sub-cultivation ratio of 1:3 in a 5% CO_2_ humidified atmosphere at 37 °C.

### 2.4. Alamar Blue Assay

RAW 264.7 cells were grown in T75 (Nunc™EasYFlask™, ThermoFisher, cat. 156472) for 2 days. After this time, fresh DMEM media containing 5% heat-inactivated FBS was added, and the cells were grown for 24 h. Cells were plated in a 96-well (flat bottom) Nunclon delta microplate (Thermo Fisher, cat.167008, Waltham, MA, USA) with a density 1 × 10^5^ cells/mL to grow for 24 h at 37 °C and 5% CO_2_ before proceeding with the assay. Then, the QD samples (Oligo, MSA, and PEG-NH_2_) were added to the cells at different concentrations (0.03, 0.06, 0.13, 0.25, 0.50, and 1.00 mg/mL), and the microplate was incubated for another 24 h. Alamar Blue reagent (Thermo Fisher, cat. DAL1025) was added (10 μL Alamar Blue per 100 μL sample, including the control wells), and incubated for 4 h at 37 °C. Colour change and increased fluorescence were quantified using absorbance at the respective excitation wavelength of 570 and 600 nm using a CLARIOstar^®^ high-performance monochromator multimode microplate reader (BMG LABTECH, Ortenberg, Germany). The Alamar Blue results were averaged over three independent experiments, with each replicate coming from a different T75 flask. Each experiment had three replicates for each well (testing compound). Finally, the reading for each plate was also done in triplicate, followed by the averaging of the values collected from the three different wells. Percentage of cell viability was calculated by following the formula [100 − ((A_0_ − A_t_)/A_0_) × 100], where, A_0_ = absorbance of cells treated with 0.1% DMSO medium, A_t_ = absorbance of cells treated with various concentrations of the samples. All results here and below were analysed using the Prism 7 software, version 7.0a.

### 2.5. Lactate Dehydrogenase Release

The LDH test-kit (PicoProbeTM LDH-Cytotoxicity Fluorimetric Assay Kit, cat. K314-500, BioVision, Milpitas, CA, USA) was used to assess the cell membrane integrity. RAW264.7 cells were collected and washed once with fresh complete growth media and plated in the 96-well microplates (5 × 10^4^ cells/well) for low control, high control, and test compounds, followed by 24 h incubation. The QD samples (Oligo, MSA, and PEG-NH_2_) were added to the cells at different concentrations (0.03, 0.06, 0.13, 0.25, 0.50, and 1.00 mg/mL), and the microplates were incubated for another 24 h. The positive control was reconstituted with 200 μL LDH assay buffer. At the end of incubation, the plate was gently shaken to ensure LDH was evenly distributed in the medium. In the high control wells, 10 μL cell lysis solution was added, and the plate was shaken for 1 min and incubated at 37 °C for 30 min. Quantitative analysis was performed on the cell culture supernatant (5 μL/well). For each well, 95 μL of LDH Reaction Mix (LDH substrate mix, PicoProbe, LDH Assay Buffer) was added, bringing up the total volume to 100 μL/well. The fluorescence (Ex/Em = 535/587 nm) was recorded using a CLARIOstar^®^ high-performance monochromator multimode microplate reader (BMG LABTECH). The medium background control reading was subtracted from all readings, and the percentage of cytotoxicity was calculated. The LDH leakage (% of positive control) was expressed as the percentage of (Test Sample − Low Control)/(High Control − Low Control), in which test samples represented the cells exposed to the QDs. The high control relates to the LDH activity in the cell (maximum LDH release control), and the low control is the LDH activity released from the untreated cells.

### 2.6. Determination of Glutothione (GSH)

RAW 264.7 cells were seeded and treated as described in preparation for Alamar Blue assay. Control cells only had complete growth medium. After 24 and 48 h of incubation with tested compounds (QDs), cell supernatants were removed and placed in −80 °C for further analysis (TNFα assay). Immediately after collecting supernatant, 400 μL was added to each well for washing and 200 μL of this was discarded. Cells were gently scraped, making a cell suspension in each well. 20 μL of the cell suspension from each well was transferred to clean Eppendorf tubes and stored at 4 °C for protein analysis. The rest of the cell suspension was treated with EDTA extraction buffer (320 μL) and incubated for 20 min at room temperature. Cell suspensions with lysed cells were transferred to clean Eppendorf tubes and centrifuged for 5 min at 4200 rpm. The supernatant was carefully collected and transferred to another tube after which 250 μL of neutralizing potassium buffer was added. The suspensions were spun again at 3500 rpm for another 5 min to obtain cell extract. Deprotonation and determination of GSH in cell extract was performed by following Glutathione assay kit (Cayman Chemical, cat. 703002, Ann arbor, MI, USA) with 50 μL of the previously obtained cell extract from different compounds and 150 μL of the assay cocktail. The plate was read for absorbance measurements after 25 min of incubation at 412 nm using a CLARIOstar^®^ high-performance monochromator multimode microplate reader (BMG LABTECH).

### 2.7. Determination of Cell Protein Content

Cell protein content was determined by using Modified Lowry Protein assay. 5 μL of cell protein sample was added to each well in triplicate in a 96-well plate. Then the mix of reagent A and B in the ratio of 1:5 in total of 200 μL was added to each well (Bio-Rad, cat. 500-0112, Hercules, CA, USA). The optical density of protein for each sample was determined by reading absorbance at 595 nm using a CLARIOstar^®^ high-performance monochromator multimode microplate reader (BMG LABTECH).

### 2.8. TNFα Assay

Expression of pro-inflammatory cytokine TNFα was determined by exposing RAW 264.7 cells treated with InP/ZnS QDs (APT, MSA, and PEG-NH_2_) with various concentrations (0.03, 0.06, 0.13, 0.25, 0.50, and 1.00 mg/mL) for 24 h and using an enzyme-linked immunosorbent assay (TNFα in vitro SimpleStep ELISA) kit provided by Abcam (ab181421, Cambridge, UK). All TNFα analysis was performed using cell supernatant followed by 24 h incubation. Each assay was performed three times, and a minimum of three wells was used as the zero control. For statistical viability, each sample was assayed in triplicate. Microplate reading (OD at 450 nm) was recorded after adding 100 μL stop solution to each well and using a CLARIOstar^®^ high-performance monochromator multimode microplate reader (BMG LABTECH).

### 2.9. ROS Production

To detect free radicals and other ROS, 1 × 10^5^ cells/well (96 well-plate, black, flat bottom) in complete growth medium were seeded and treated using the phagocytosis assay through the combination of two specific fluorescent probes. Cellular ROS/Superoxide detection assay kit (Abcam, ab139476) was chosen to provide the real-time measurement of global levels of ROS, as well as superoxide concentrations in living cells, specifically. After 48 h, the culture medium was removed and changed with fresh media. For the negative control, ROS inhibitor (N-acetyl-L-cysteine) was added to pre-treat the cells for 30 min beforehand. Then the cells were loaded and incubated with the ROS/superoxide detection mix (1× wash buffer), oxidative stress detection reagent (green fluorescence), and superoxide detection reagent (orange fluorescence) for 30 min at 37 °C. After washing, oxidative stress (ROS, green fluorescence) and superoxide (red fluorescence) positive cells were analysed using an Olympus IX53 (Tokyo, Japan) inverted microscope with the recommended filter set for oxidative stress detection compatible with fluorescein (Ex/Em = 490/525 nm) and superoxide detection filter set compatible with rhodamine (Ex/Em = 550/620 nm). GSH measurements were performed according the formula Glutathione reductase (GR) = (ΔB / (T_2_ − T_1_) × 0.9 × V) × Sample Dilution Factor, where ΔB is taken from the standard curve (moles), T_1_ is the time of the first reading (A_1_) in min, T_2_ is the time of the second reading (A_2_), V is the volume added into the reaction well (mL), and 0.9 is the sample volume change factors during the sample pre-treatment procedure. Absorbance (OD) was immediately measured at 405 nm for all readings. The reaction was incubated for 10 min at room temperature between readings.

### 2.10. Fluorescence Microscopy

RAW 264.7 cells were seeded at 1 × 10^5^ cells/well in a Thermo Scientific™ Nunc™ F96 MicroWell™ Black Polystyrene Plate (cat. 137101) and incubated with different surface chemistry QDs (Oligo, MSA, and PEG-NH_2_) at a concentration of 1 mg/mL for 2 h at room temperature and placed on a bidirectional rotating plate. After the incubation step, each well was washed with 1 × PBS to remove unbound QDs. Nuclei were labelled with Hoechst 33342 (Thermo Fisher, cat. H1399) and viewed immediately. Live cell imaging with the QDs was performed using 596 nm laser excitation and a 615 nm emission filter for red fluorescent QDs, and the laser set with Ex/Em = 346/460 nm for nuclear staining. Live cell observation, image capture, and fluorescence observation were performed with an Olympus IX53 inverted microscope.

### 2.11. Synthesis of InP/ZnS QDs

InP/ZnS QDs were synthesised by a method adapted by Tessier et al [16]. Indium(III) chloride (0.45 mmol), zinc(II)chloride (2.2 mmol and 5.0 mL of oleylamine were mixed under vacuum and heated to 120 °C under vacuum for 60 min. The mixture was then heated to 180 °C while under nitrogen. After this temperature was reached, tris-(diethylamino)phosphine (1.6 mmol) was rapidly injected into the mixture. After 20 min, sulphur in trioctylphosphine (TOP-S) (1 mL, 2.2 M) was injected over 10 min. After 60 min, the temperature was ramped to 200 °C, and after 120 min, 1 g of Zn(stearate)_2_ in 4 mL of 1-octadecene (ODE) was introduced dropwise (over 10 min). The temperature was increased to 220 °C. At 150 min, 0.7 mL of TOP-S was injected slowly. The temperature was increased to 240 °C. At 180 min, 0.5 g of Zn(stearate)_2_ in 2 mL of ODE was added, and the temperature was increased to 260 °C. After 210 min, the reaction ended, and the reaction mixture was diluted with toluene. The resulting QDs were precipitated in ethanol and resuspended in toluene. Size exclusion chromatography was done as a final purification step, yielding red-emitting (~600 nm) InP/ZnS QDs.

### 2.12. Ligand Exchange

For InP/ZnS MSA QDs, 0.30 g of MSA was mixed with 1 mL of toluene and left to stir (10–15 min). 1 mL of QDs (10 mg/mL) was added to the cloudy mixture and stirred for 1 min. Subsequently, 1 mL of ammonium hydroxide and 1 mL of Milli-Q water were added and left to stir overnight. The red aqueous layer was purified by precipitating in ethanol and centrifuging. The pellet was re-dispersed in 2 mL of Milli-Q water and the resulting water-soluble InP/ZnS-MSA QDs were stored in the dark at −4 °C [17].

For InP/ZnS PEG-NH_2_, 10 mg of SH-PEG-NH_2_ was dissolved in 2 mL of chloroform. This was mixed with 100 µL of a 10 mg/mL QD solution in chloroform and was stirred overnight. The QDs were then washed with 5mL of hexane and then with a mixture of ethanol and hexane (3:5). After centrifuging at 10,000 rpm for 10 min, the QDs were re-dispersed in 1 mL of Milli-Q water [18].

For InP/ZnS Oligo, 5 µL of 1 mg/mL Oligo was added to QD-MSA (1 mg/mL) and stirred. 1-Ethyl-3-(3-dimethylaminopropyl)carbodiimide (EDC; 0.02 M) and N-hydroxysuccinimide (NHS; 0.05 M) were added and allowed to stir for 2 h. The QD-Oligo was washed using a 30kDa Amicon filter and centrifuged twice at 19,000 rpm for 15 min. The QDs were re-dispersed in 1 mL of Milli-Q water. The oligonucleotide was purchased from DNA technologies (IDT) and had the following sequence: 5AmMC12/5’-CA CCC CAC CTC GCT CCC GTG ACA CTA ATG CTA-3’.

### 2.13. Characterisation of InP/ZnS QDs

The size and shape of the QDs were determined using transmission electron microscopy (TEM). The analysis was conducted using a 200 KV JEOL 2100F (JEOL, Tokyo, Japan). For each synthesised InP/ZnS QDs, 5 µL of sample was pipetted onto carbon-coated 300-mesh copper grids. After 30 min of adsorption, the excess suspension was removed using filter paper, and the sample was left to dry completely overnight. Steady-state photoluminescence measurements on InP/ZnS QDs in the range 520–800 nm were acquired using the FLS-980 Photoluminescence spectrometer. Quantum yields were calculated using the integrating sphere. The samples were excited at 480 nm. The sizes were measured using Gatan Microscopy Suite Software (GMS 3, Pleasanton, CA, USA). Dynamic light scattering (DLS) was done using Malvern Zetasizer ZS90 to measure the hydrodynamic diameter and zeta potential (ZP) of the QDs.

## 3. Results

### 3.1. Synthesis and Characterisation of InP/ZnS QDs

**Figure 1:**
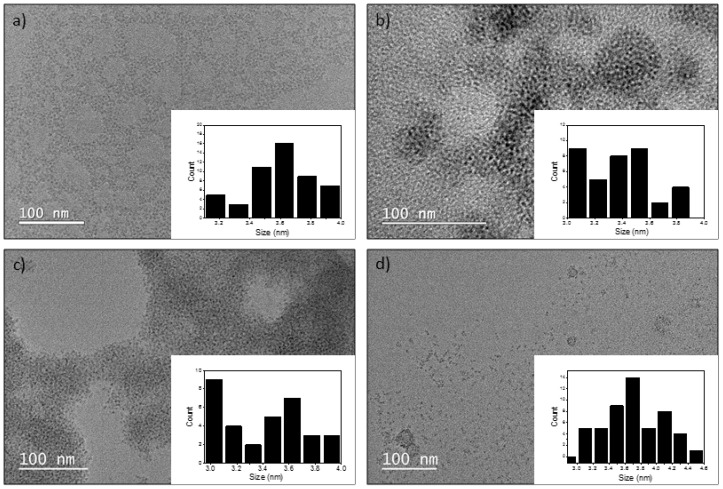
Transmission electron microscope images of QDs with different functionalities with size histogram (inset); (**a**) InP/ZnS- Oleylamine (**b**) InP/ZnS-PEG-NH_2_ QDs (**c**) InP/ZnS-Oligo (**d**) InP/ZnS-MSA QDs.

The synthesis of InP/ZnS QDs was based on a method developed by Tessier et al., where aminophosphine precursors were used as a safer alternative [16]. The purified InP/ZnS QDs had a further ligand exchange with different compounds (MSA, Oligo, and PEG-NH_2_) to make them water soluble and more functional for biomedical applications (Figure 1). A summary of the physical and chemical characterisation of each QD is shown in Table 1, including the photoluminescent properties, TEM images to measure diameter and DLS to measure hydrodynamic radius.

PEG-NH_2_ functionalised QDs have a larger hydrodynamic diameter (59 nm) as measured using DLS, compared to the MSA-functionalised and Oligo-QDs. DLS measures the hydrodynamic size through the measurement of the Brownian motion of the particles, which is then related to size. The Brownian motion is due to the movement of the particles and the interaction of the solvent molecules around the particle. As a result of this, the hydrodynamic diameters include the ligand and the solvent environment around the particle. The resulting larger diameter obtained for the PEG- NH_2_ QDs is therefore explained, as the PEG is a large, polymeric ligand compared to the smaller Oligo and small-molecule MSA. The MSA-QDs are larger than the Oligo-QDs, and this could further be due to the wrapping of the Oligo around the nanoparticle. According to the diameter as measured by TEM, InP/ZnS QDs with different surface chemistries showed similar results with small variance in the mean size (3.5–3.6 nm), as well as emission spectra (600–604 nm). The TEM results show small changes in the mean size due to only the QD core and shell being visible under the microscope. The three ligands used are all carbon-based and will not be visible using TEM due to the use of a carbon-coated grid for imaging. Further to this, the low contrast of the materials introduces inaccuracies in the measurement of the size, and therefore, the size distributions are broad. ZP, defined by intensity of QDs, was higher, interestingly, for Oligo, followed by PEG-NH_2_ and MSA, respectively, which can be attributed to the charge of the different molecules, as well as the stability of the particles in water. Overall, DLS measures a significantly larger population of nanoparticles in solution, which further means that several large aggregates that could be present in solution are also included in the measurement of size. These are further visible in TEM (Figure 1b–d), and these particles were not measured when measuring diameter for obtaining size. Hence, the hydrodynamic diameter of nanoparticles is greater than the estimated size by TEM.

### 3.2. Alamar Blue and LDH Assay

**Figure 2:**
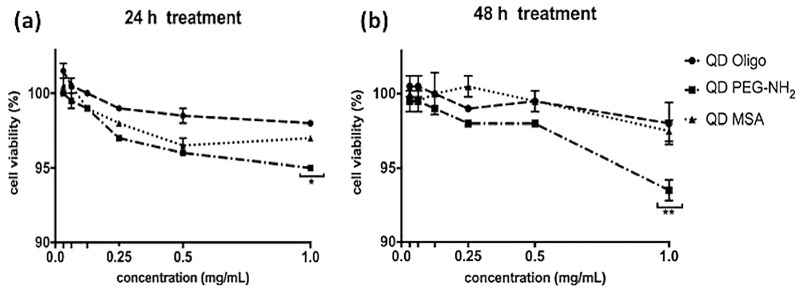
In vitro cytotoxicity of RAW 264.7 macrophage cells treated with QDs with different surface chemistries (Oligo, PEG-NH_2_, and MSA). The graph represents cell viability at (**a**) 24 h and (**b**) 48 h, determined by Alamar Blue assay. Data represents mean standard error of the mean (*n* = 3; * (*p* < 0.05), and ** (*p* < 0.01); one-way ANOVA).

The metabolic activity was measured using resazurin (Alamar Blue assay), as shown below (Figure 2). Cell viability was measured on RAW 264.7 at 24 h and 48 h after exposure to QDs with Oligo, PEG-NH_2_ and MSA functionalities in a range of concentrations (0.03, 0.06, 0.13, 0.25, 0.50, and 1.00 mg/mL).

According to the collected data, cell viability was above 95% 24 h post treatment and above 90% 48 h post treatment for all functionalized QDs for all chosen concentrations. Hence, the best performance showed Oligo and MSA QDs for both treatment time and concentration.

**Figure 3:**
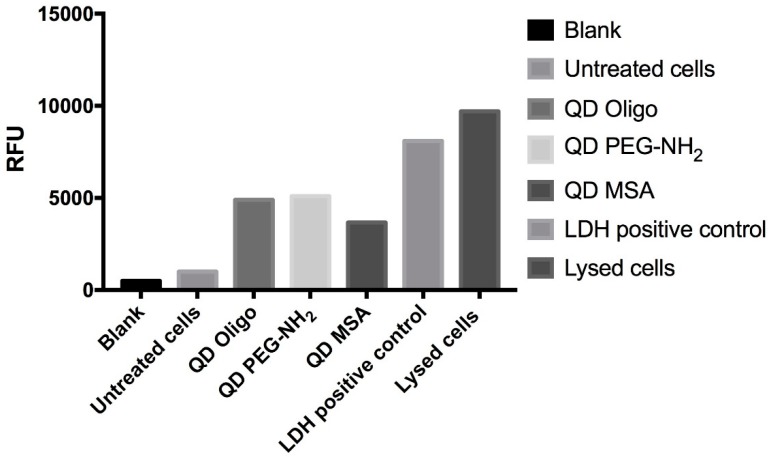
Mean of LDH release from RAW 264.7 cells treated with QDs with ligands Oligo, PEG-NH_2_ and MSA at 24 h (48 h is not present) (*n* = 3). Data analysis is expressed as the % mean compared to untreated cells, positive control (0.1% Triton X-100), and lysed cells.

**Figure 4:**
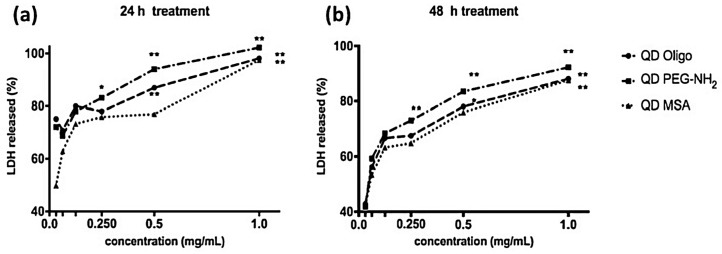
Percentage LDH release from RAW 264.7 cells treated with QDs with Oligo, PEG-NH_2_ and MSA ligands at (**a**) 24 and (**b**) 48 h (*n* = 3). Data is expressed as the % mean compared to positive control (0.1% Triton X-100) ± standard error of the mean. * (*p* < 0.05) and ** (*p* < 0.01) indicate significant differences compared to control cells treated with completed medium only.

The LDH release (Figure 3 and Figure 4) was used as a complementary assay to the metabolic activity assay, as it investigates the membrane integrity. Overall, there was a significant increase in LDH release from RAW 264.7 cells at concentrations above 0.25 mg/mL (Figure 4). Both the QD PEG-NH_2_ and QD Oligo samples showed a significant increase (~80–100%) at higher concentrations (*p* < 0.01 above 0.25 mg/mL). However, the QD MSA only showed a significant increase at 1 mg/mL (*p* < 0.01) (Figure 4).

### 3.3. ROS Production

**Figure 5:**
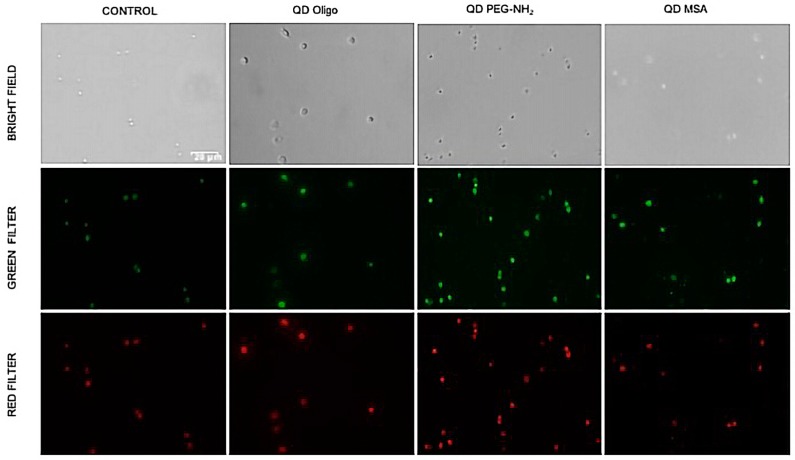
Profiling of ROS formation by fluorescence microscopy was achieved in RAW 264.7 cells loaded with ROS/Superoxide detection reagents and treated with QDs (Oligo, PEG-NH_2_, and MSA). Control (untreated) samples indicate low green and red fluorescence upon induction. General oxidative stress levels were monitored in the green channel, while superoxide production was detected in the red channel. Scale bar: 20 μm.

ROS generation was measured using ROS-sensitive fluorescence dyes (Figure 5) with the highest concentration of InP/ZnS QDs (1 mg/mL; Oligo, PEG-NH_2_, and MSA). There were two fluorescent probes: one indicative of cellular production of ROS (green fluorescence) and another superoxides (O^2−^, red fluorescence). As shown (Figure 5), ROS and superoxide production were observed with the emission of the fluorescent probes. This observation is in line with a previous report demonstrating that cellular response can be greatly influenced by the surface ligands, rather than the composition of the nanoparticles, finding that PEG-functionalised QDs were more cytotoxic than those functionalised with mercapto acids [19].

**Figure 6:**
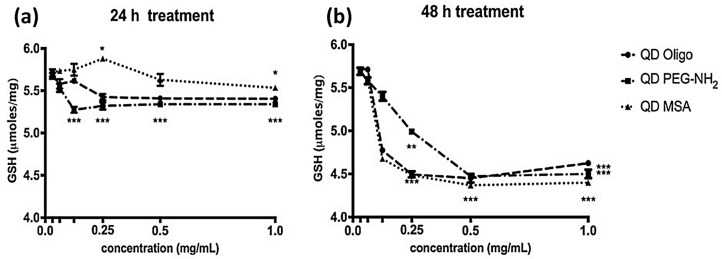
GSH protein levels in RAW 264.7 cell extracts after treatment with QDs Oligo, PEG-NH_2_, and MSA for (**a**) 24 h and (**b**) 48 h (*n* = 2). Data is presented as mean (μmoles/mg) SEM with * (*p* < 0.05), ** (*p* < 0.01), and *** (*p* < 0.001).

GHS reacts specifically with O-Phthalaldehyde and can be directly related to the GSH concentration. Therefore, the dependence of glutathione production upon QD exposure was investigated. For that purpose, the intracellular GSH levels of the cell model (RAW 264.7) were measured at different treatment times (24 h and 48 h) [20]. Results are presented in Figure 6 for 24 and 48 h post treatment. After 24 h, QD PEG-NH2 showed a slight decrease in GSH levels (5.3 μmoles/mg), and after 48 h, there was a decrease in GSH (4.5 μmoles/mg) for all QDs.

### 3.4. Fluorescence Microscopy of Cells Exposed to QDs

**Figure 7:**
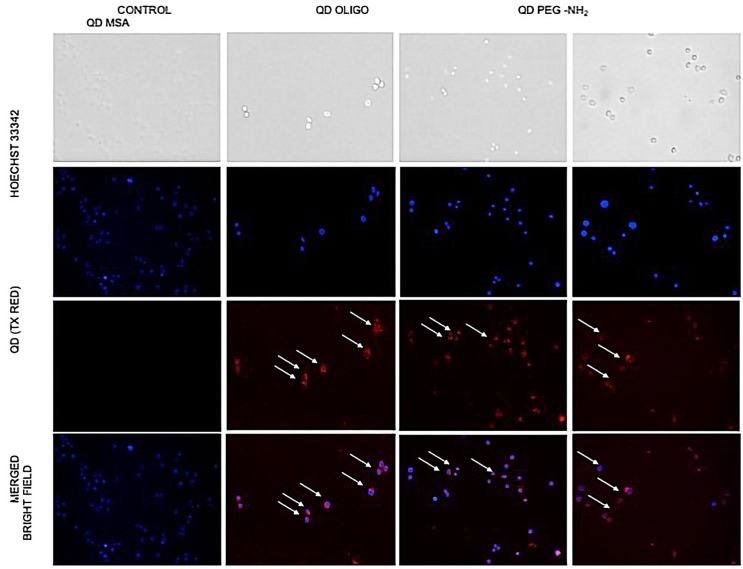
Fluorescence microscopy of RAW 264.7 cells exposed to QDs Oligo, QD PEG-NH_2_ and QD MSA after 2 h treatment. The cell nucleus was stained with HOECHST 33342 (blue). The signals from QDs are shown in red. The arrows show co-localization of nucleus and QDs. Scale bar: 20 μm.

Cellular uptake of each QDs (Oligo, MSA, and PEG-NH_2_) at 1 mg/mL was observed using fluorescence spectroscopy (Figure 7). As shown in Figure 7, below, QD PEG-NH_2_ (third column) appeared distributed around the nuclei, as the red fluorescence (QD) overlapped with the blue fluorescence (nuclei staining). Meanwhile, the QD MSA (fourth column) and QD Oligo (second column) images showed evidence of membrane-attachment of the nanoparticles compared with the control group. Also, lower InP/ZnS MSA QDs intake was observed compared to QD PEG-NH_2_ and QD Oligo.

## 4. Discussion

Cadmium-based QDs have long been used for bio-imaging applications; however, concerns related to exposure to the toxic heavy metal have led researchers to search for other alternatives, such as InP/ZnS QDs, due to the lower toxicity of indium. Studies related to the toxicity of InP/ZnS QDs are scarce, with Chibli et al. examining the ROS generation and concluding a lower toxicity for InP when compared to Cd-based alternatives [12]. Chen et al. investigated the effect of InP/ZnS QDs with differently terminated polymer ligands on a lung-derived cell line and found higher toxicity of COOH- and NH_2_-terminated QDs as compared to hydroxyl-terminated ones [21]. This work further looked at different ligand environments by extending the variety of possible ligands and surface termination that can be used for biomedical applications. Therefore, mercaptoacid ligand (MSA) and HS-PEG-NH_2_ were used to investigate both the effects of small and larger polymer molecule ligands respectively, as well as –COOH and amine termination. These two ligands are popular functionalities for use in biological applications, as surface modification for solubilisation of QDs in aqueous environments is a necessary step. An oligonucleotide was also used to investigate further cytotoxicity of the bioconjugate. The functionality of the QDs can be modified through conjugation to other biomolecules using terminating groups such as carboxyl or amine for cross-linking. Upon ligand exchange of the as-prepared QDs, minor emission maximum shifts were observed in InP/ZnS QDs, with a decrease in quantum yields, demonstrated by decreased emission intensity [22]. This is common with ligand exchange, as the integrity of the QD is decreased when exposed to solvents [23].

Raw 264.7 murine “macrophage-like” cells were chosen as an in vitro cell culture model to examine cytotoxic effects of InP/ZnS QDs. The choice of the cell model was based on the expected anti-inflammatory response and subsequent production of cytokines when exposed to nanoparticles. It is well known that macrophage cells play a significant role in immune responses as they scavenge and initiate defence mechanisms in conjunction with other immune cells, such as lymphocytes. Moreover, Clemens et al. showed that macrophages have a greater capacity to “internalise” nanoparticles, thus making macrophage cells an ideal candidate for cytotoxic analysis [24]. Figure 3 showed no significant levels of cytotoxicity at high concentrations (1 mg/mL), showing 95% cell survival for all QDs (QD MSA, QD PEG-NH2 and QD Oligo) after 24 h treatment and over 90% for all QDs after 48 h of treatment. Overall, QD PEG-NH_2_ showed the highest cell survival rates compared to the cells exposed to QD MSA and QD PEG-NH_2_. The cytotoxicity of InP/ZnS QDs was substantially lower compared to cadmium-based nanomaterials with various chemistries, with organic-capped CdTe/CdSe having 20% viability and those functionalised with PEG-NH_2_ moieties having 40% at the highest concentrations [5].

LDH is a complimentary metabolic activity assay that showed an increase of LDH (100%) at the highest concentrations of QDs (1.0 mg/mL) after 24 h. After 48 h, an increase of LDH release was measured at 90% (Figure 3 and Figure 4). Overall, no significant membrane disruption was observed upon QD exposure after 24 h and 48 h. Comparatively, Clift et al. showed that LDH release was substantially higher for cells exposed to cadmium-based QDs with surface functionalities (up to 120%) [25]. The detection of TNFα was done to observe pro-inflammatory responses of QDs with different surface chemistries and concentrations (Figure S2). Even at the highest concentrations (1.0 mg/mL), the QD Oligo, QD PEG-NH_2_ and QD MSA had no pro-inflammation response in comparison to the control samples.

Profiling of ROS formation after 24 h of incubation with QD samples, the level of GSH produced by the cells decreased in most cases, with a maximum reduction of ~10% observed by cells exposed to QD PEG-NH_2_ samples. QD-MSA exhibited the least effect on antioxidant production and was seen to raise production levels above those of the control for 0.25 mg/mL concentrations (*P* < 0.05). After 48 h treatment, GSH production was further inhibited, resulting in a maximum of 20% decrease for the high-concentration samples (*p* < 0.001). Overall, all QD samples showed similar a similar cellular GSH production. For the samples with concentrations ≤0.25 mg/mL in this time frame, QD PEG-NH_2_ exhibited the least effect on antioxidant production, which is contrary to Figure 6a. It was also reported that there was an increased lysosomal activation in HeLa cells that coincided with increased ROS generation, but further work may be needed to verify that this also occurs with InP-based QDs. Data in Figure S1 represents positive (induced with ROS inducer Pyocyanin) and negative (pre-treated with ROS inhibitor N-acetyl-L-cysteine) controls and QDs treated with 2× ROS/Superoxide Detection Mix. It is possible that QDs themselves generate ROS and affect cell signalling, including their growth, cellular uptake or lysosomal production. Further studies are needed to observe the rate of cellular update and related ROS production. Cadmium telluride and selenide (CdTe and CdSe) QDs with a zinc sulphide (ZnS) shell exposed to J774.A1 macrophage cell lines in other cytotoxicity studies showed a similar response in terms of the ligand sensitivities [5]. When comparing the cellular response shown here, InP/ZnS have an overall lower cytotoxic effect compared to previously reported data shown for CdTe/ZnS, as expected [5].

Fluorescent microscopy (Figure 7) was used to observe changes in cell morphology, and cellular uptake occurred. No changes in cell morphology was observed, but there was a difference in uptake levels with QD MSA showing the lowest cell uptake. Chen et al. demonstrated that InP/ZnS QDs resulted in an increased ROS production with prolonged exposure and cellular uptake with the lung-derived cell line [21]. Comparatively, there is evidence of this with this work, but further investigation is required.

## 5. Conclusions

In summary, this study demonstrated that InP/ZnS QDs with different chemical surfaces (Oligo, PEG-NH_2_, and MSA) have low cytotoxic behaviour after 24 and 48 h exposure, compared with cadmium-based QDs [5]. Similarly, the TNFα production indicates that there was no pro-inflammation response to the QDs. With that said, there was an increase in LDH release, which confirms that there was some membrane disruption with prolonged exposure of 24 and 48 h. This complements the increased ROS production, which was seen after 48 h, and has been reported to be observed with cellular uptake. The membrane disruption indicated by the LDH release gives some evidence that there may be an increase in lysosome generation. Further investigation into ROS production and the lysosomal production (including exosomes) is needed to substantiate this hypothesis.

Conjugating the surface of InP/ZnS QDs with ligands is vital for their customised use in bioimaging and therapeutic applications. With that said, different chemical surfaces, in particular PEG-NH_2_ compared to MSA, demonstrated different cellular responses and need to be taken into consideration. Overall, InP/ZnS QDs conjugated with Oligo, MSA or PEG-NH_2_ can be considered a good alternative to other more toxic QDs (e.g., cadmium and lead) but further analysis of long-term effects (DNA damage) and lysosomal activities after QD exposure is needed.

## Figures and Tables

**Table 1:** Summary of the properties of the InP/ZnS QDs obtained using PL (photoluminescent emission), TEM and dynamic light scattering to determine QD diameter and hydrodynamic radius, respectively.

	InP/ZnS-Oligo	InP/ZnS PEG-NH_2_	InP/ZnS MSA
Emission maximum (nm)	600	605	604
Hydrodynamic diameter (nm)	21	59	38
ZP (−mV)	41.9	32.6	31.9

## Supplementary Materials

The following are available online at http://www.mdpi.com/2079-4991/9/2/135/s1, Figure S1: Positive and negative controls of ROS; Figure S2: Representation of TNFα secretion on RAW 264.7 cells vs concentration, after treatments with QDs with ligands Oligo, PEG-NH_2_ and MSA are available free of charge via the Internet at http://pubs.acs.org.

## Abbreviations

Zinc sulphide (ZnS), tumour necrosis factor alpha (TNFα), glutathione (GSH), polyethylene glycol (PEG), reactive oxygen species (ROS), lactate dehydrogenase (LDH).
