# Supplementary material for: An In Vitro Investigation of Cytotoxic Effects of InP/Zns Quantum Dots with Different Surface Chemistries
Source: Nanomaterials (Basel). 2019 Jan 22;9(2):135. doi: 10.3390/nano9020135 (PMC6409980; doi:10.3390/nano9020135)
Supplement: Supplementary file 1 [file nanomaterials-09-00135-s001.pdf]

nanomaterials

An In Vitro Investigation of Cytotoxic Effects of InP/Zns Quantum Dots with Different Surface Chemistries

Deanna Ayupova [1,2,3], Garima Dobhal [1,2,3], Geoffry Laufersky [1,2,3], Thomas Nann [1,3,†] and Renee V. Goreham [1,2,3,*]

[1] MacDiarmid Institute for Advanced Materials and Nanotechnology, Wellington 6140, New Zealand; deanna.ayupova@vuw.ac.nz (D.A.); garima.dobhal@vuw.ac.nz (G.D.); geoffry.laufersky@vuw.ac.nz (G.L.); thomas.nann@newcastle.edu.au (T.N.)
[2] Centre for Biodiscovery, Victoria University of Wellington, Wellington 6012, New Zealand
[3] School of Chemical and Physical Sciences, Victoria University of Wellington, Wellington 6012, New Zealand
* Correspondence: Renee.Goreham@vuw.ac.nz
† Current address: School of Mathematical and Physical Sciences, University of Newcastle, Newcastle 2304, Australia

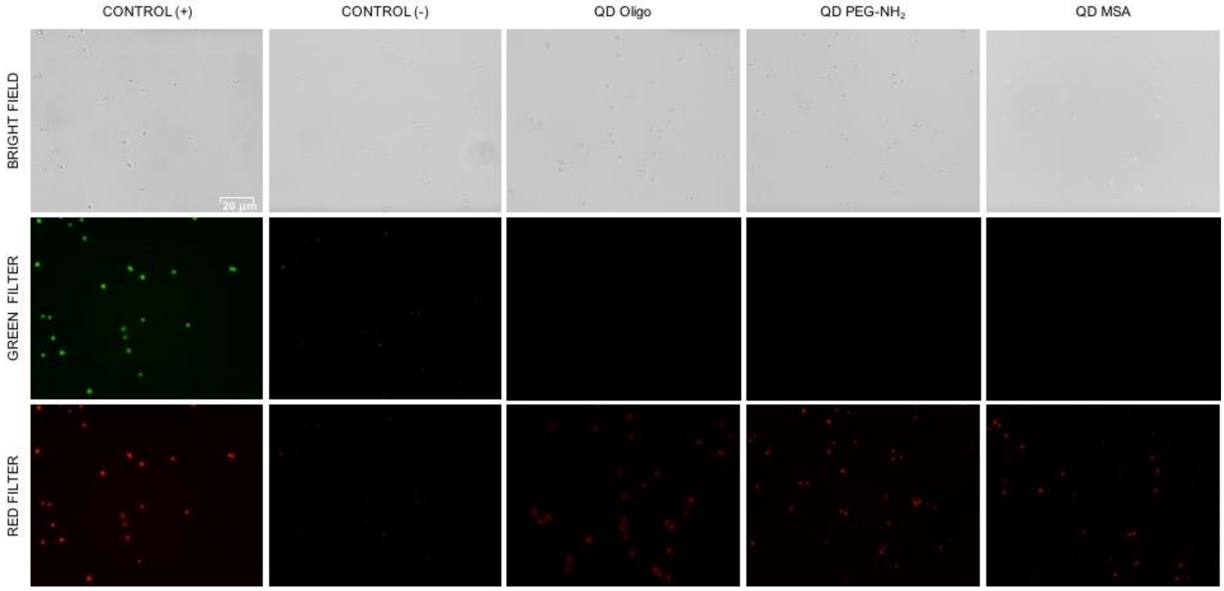

Figure S1. Data represents positive (induced with ROS inducer Pyocyanin) and negative (pre-treated with ROS inhibitor N-acetyl-L-cysteine) controls and QDs treated with 2x ROS/Superoxide Detection Mix. As a result, positive control exhibit both bright green and red fluorescence, negative control shows no significant green or red fluorescence upon induction, and QDs present only in red channel.

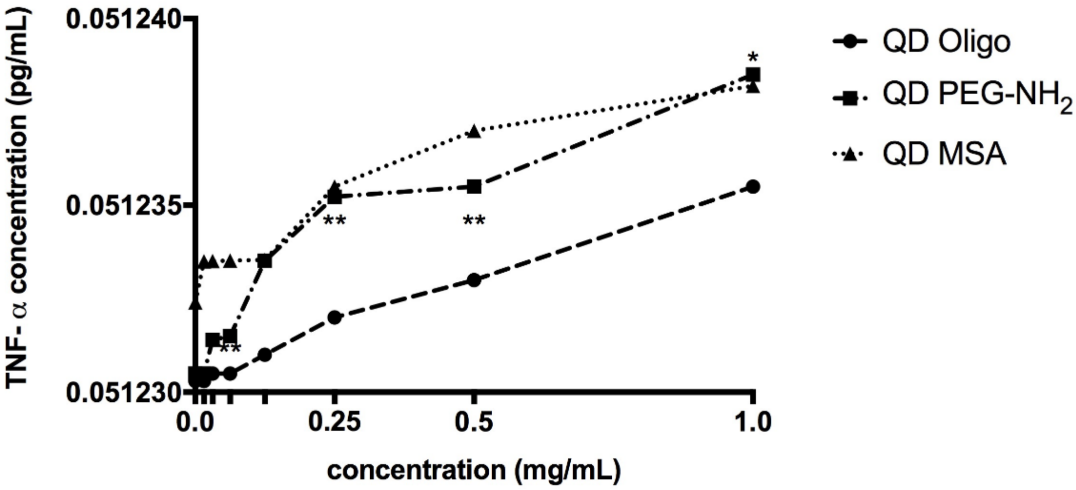

Figure S2. Representation of TNFα secretion on RAW 264.7 cells vs concentration, after treatments with QDs with ligands Oligo, PEG-NH_2 and MSA. Data represents mean SEM (*n* = 3).
